# Ershen Dan attenuates atherosclerosis by modulating the NOTCH1/NF-κB/NLRP3 signaling pathway to suppress M1 macrophage polarization

**DOI:** 10.1186/s13020-025-01232-9

**Published:** 2025-10-23

**Authors:** Wenjie Zhao, Hui Wang, Shijing Peng, Qing Wei, Lei Zhang, Yunlun Li, Wenqing Yang

**Affiliations:** 1 Innovation Research Institute of Traditional Chinese Medicine, Shandong University of Traditional Chinese Medicine, Jinan, 250300 China; 2 Key Laboratory of Traditional Chinese Medicine Classical Theory, Ministry of Education, Shandong University of Traditional Chinese Medicine, Jinan, China; 3 Department of Cardiovascular, Affiliated Hospital of Shandong University of Traditional Chinese Medicine, Jinan, 250014 China; 4 Shandong Province Engineering Research Center for Precision Diagnosis and Treatment of Cardiovascular Diseases in Chinese Medicine, Jinan, 250300 China

## Abstract

**Background:**

Atherosclerosis is a chronic inflammatory disease. Inhibition of macrophage inflammatory secretion is the key to the prevention and treatment of atherosclerosis. Ershen Dan (ESD) has been shown to be effective in treating atherosclerosis; however, its pharmacological mechanisms remain unclear. This study elucidated the mechanism of action of ESD by investigating its relationship with macrophages.

**Materials and methods:**

The chemical composition of ESD was analyzed using ultra performance liquid chromatography (UPLC)-Q-Exactive-tandem mass spectrometry (MS/MS). In the *in vivo* experiments, serum levels of low-density lipoprotein (LDL), triglycerides (TG), and total cholesterol (TC) in ApoE^−/−^ mice were measured using a biochemical analyzer. The serum levels of key inflammatory factors were quantified using an enzyme-linked immunosorbent assay (ELISA). The aortic lipid plaque area was assessed using Oil Red O staining, while plaque characteristics were evaluated using hematoxylin and eosin (H&E), Masson trichrome, and Sirius Red staining techniques. Network pharmacology analyses in conjunction with molecular dynamics simulations was used to screen the active components of ESD and their target proteins. By integrating database resources, the key target genes related to inflammatory responses in atherosclerosis were identified. The expression levels of neurogenic locus notch homolog protein 1 (NOTCH1), hairy and enhancer of split-1 (HES1), NOD-, LRR- and pyrin domain-containing protein 3 (NLRP3), and nuclear factor (NF)-κB in mouse aortic tissue were detected using western blot analyses. CD86 levels in mouse aortas were quantitatively analyzed by immunohistochemistry. In the *in vitro* experiments, RAW264.7 cell viability was assessed using the Cell Counting Kit-8 (CCK-8) assay. Macrophage phenotypic changes were evaluated using immunofluorescence analyses. Intracellular levels of reactive oxygen species (ROS) were measured using dichloro-dihydro-fluorescein diacetate (DCFH-DA) probes. The expression levels of key proteins and genes were validated by western blotting and quantitative real-time polymerase chain reaction (qRT-PCR).

**Results:**

Twenty bioactive chemical components were identified in ESD. *In vivo* studies demonstrated that ESD inhibits macrophage secretion of inflammatory factors through the NOTCH1/NF-κB/NLRP3 signaling pathway, indicating its therapeutic potential for atherosclerosis. *In vitro* studies further revealed that ginsenoside Rg_1_ and tanshinone II_A_, which are active constituents of ESD, exert anti-inflammatory effects by suppressing the NOTCH1/NF-κB/NLRP3 pathway and reducing intracellular ROS levels in macrophages, supporting their role in atherosclerosis treatment.

## Introduction

Cardiovascular diseases are a leading cause of death worldwide. Atherosclerosis is the core pathological basis for these conditions, and it imposes substantial health and economic burdens [1]. In China, cardiovascular disease accounts for approximately half of all deaths, and atherosclerosis-induced vascular obstruction has been identified as the primary underlying cause in these cases [2, 3]. The pathogenesis of atherosclerosis involves complex mechanisms, which are primarily driven by dyslipidemia and chronic inflammation [4, 5]. During disease progression, macrophages play crucial dual roles: proinflammatory M1 macrophages mediate endothelial damage and contribute to plaque instability, whereas anti-inflammatory M2 macrophages promote plaque stabilization [6, 7]. The existing first-line pharmacological interventions for atherosclerosis primarily function through lipid-lowering and anti-inflammatory effects [8]. However, these agents are associated with risks of hepatic and renal toxicity [9]. Consequently, the development of novel therapeutic agents for atherosclerosis is an urgent priority.

Traditional Chinese medicine (TCM) offers unique advantages for the treatment of atherosclerosis. A variety of preparations, such as Compound Danshen Dropping Pills, Tongxinluo Capsules, and Xuezhikang Capsules, have been developed and widely used in clinical practice with good therapeutic effects [10, 11]. *Panax ginseng* C.A. Mey and *Salvia miltiorrhiza* Bge are two of the primary constituents of Ershen Dan (ESD), an empirical formula for the treatment of stable coronary heart disease in the Department of Cardiology of the Affiliated Hospital of Shandong University of Traditional Chinese Medicine. This formula has been used in clinical practice for many years and has shown definite efficacy and the ability to tonify qi and promote blood circulation. *Panax ginseng* C.A. Mey, which is used in this prescription, is a member of the Araliaceae family. The dried roots and rhizomes of this plant are known to greatly replenish vital energy, benefit the spleen and lungs, and promote the production of body fluids and nourishment of blood. *Salvia miltiorrhiza* Bge, another constituent of ESD, belongs to the Lamiaceae family. However, the specific mechanisms through which ESD exerts its anti-atherosclerotic effects remain unclear. Therefore, this study employed modern pharmacological and molecular techniques to investigate the connections between ESD and macrophage modulation and their underlying mechanisms. Furthermore, we aimed to identify the active components primarily responsible for the therapeutic effects of ESD, providing a scientific foundation for the use of this formulation in the treatment of atherosclerosis.

## Materials and methods

### Drug preparation

ESD consisted of two drugs (Table 1). The raw materials of the Chinese medicines used in this study were purchased from the Affiliated Hospital of Shandong University of Traditional Chinese Medicine (Jinan, China). Appropriate amounts of ginseng and danshen were soaked 10 times in 70% ethanol for 30 min, sonicated for 60 min, and filtered; subsequently, 70% ethanol was added five times, and the solution was sonicated for 30 min. After filtration, the filtrates were combined, concentrated by rotary evaporation, and freeze-dried.

**Table 1:** Prescription of ESD

Local name	English name	Latin name	Part used	Origin (P. R. China)	Amount (g)
Ren Shen	Ginseng	*Panax ginseng* C. A. Meyer	Root	Ji Lin	9
Dan Shen	Salvia miltiorrhiza	*Salvia miltiorrhiza* Bge	Root	An Hui	15

### Preparation and composition analysis of the ESD standard solution

The ultra performance liquid chromatography (UPLC)-tandem mass spectrometry (MS/MS) analysis was conducted using the Ultimate 3000-Q-Exactive system (Thermo, USA). The chromatographic conditions were as follows: chromatographic column, ACQUITY UPLC HSS T13 (2.1 mm × 100 mm, 1.8 μm); mobile phase, acetonitrile solution (A) and 0.05% phosphoric acid solution (B); gradient elution conditions, 10–19% B over 0–8 min, 19–50% B over 8–35 min, 50–100% B over 35–40 min, 100–19% B over 40–42 min, and 19–19% B over 42–45 min; flow rate, 3 μL/min; injection volume, 250 μL; and column temperature, 30 °C. For mass spectrometry analyses, collection was conducted in the positive- and negative-ion modes, with the first and second-stage mass scans in the range of 80–1200 m/z and 200–2000 m/z, respectively, and collision energies of 20, 40, and 60 eV.

### Animal model

In this study, an atherosclerosis model was established in ApoE^−/−^ mice, and C57BL/6 J mice were used as blank controls. All mice were 6–7 weeks old, male, and weighed 18–20 g (Beijing Weitonglihua Co., Ltd.). After obtaining approval from the Experimental Animal Ethics Committee of Shandong University of Traditional Chinese Medicine, the animals were raised in the Animal Experiment Center (Ethics Number: SDUTCM20240103001). All mice were maintained on a 12-h light/dark cycle every day and provided free access to food and water. ApoE^−/−^ mice were fed atherosclerotic model feed for 12 weeks to establish the atherosclerosis model. These mice were randomly divided into six groups according to body weight, with eight mice in each group. Rosuvastatin (RSV) (505286; Ariscan Pharmaceuticals Ltd., China) was used as the positive control and was administered at a dose of 10 mg/kg/day [13]. C57BL/6 J mice administered the same dose of phosphate-buffered saline (PBS) were considered as the control group. The drug treatment groups included the Er Shen Dan high-dose (ESD-H) group, in which mice were administered a dose of 6.24 g/kg/d; the Er Shen Dan medium-dose group (ESD-M), in which mice received a dose of 3.12 g/kg/d; and the Er Shen Dan Low low-dose group (ESD-L), in which mice were administered a dose of 1.56 g/kg/d. The drugs in the treatment groups were administered by gavage for 8 weeks. After the experiment, the mice were anesthetized with isoflurane (R510-22-10; RWD, China), and their serum was collected. After cardiac perfusion, the aorta was removed for the subsequent experiments.

### Blood biochemical tests

The prepared serum samples were placed in the sample chamber (URIT-8026) of the analyzer, and the reagents required for testing were loaded. Low-density lipoprotein (LDL) (U83085045, URIT, China), triglyceride (TG) (U82781050, URIT)) and total cholesterol (TC) (U82885040, URIT) levels were measured, and the results were analyzed.

### Enzyme-linked immunosorbent assay

Mouse blood was centrifuged at 3500 rpm and 4 °C for 15 min. The serum was stored at −80 °C. The tumor necrosis factor-α (TNF-α), interleukin (IL)−1β, and IL-6 levels in the serum of the mice were detected according to the instructions of the enzyme-linked immunosorbent assay (ELISA) kits. The MF-Mouse TNF-α ELISA Kit (RE1060MF), Mouse High Sensitivity IL-1β ELISA Kit (RE1074MG), and the MF-Mouse IL-6 ELISA Kit (RE3186MF) were purchased from Milbio Company (Shanghai, China).

### Oil Red O staining

Complete blood vessels from the aortic arch to the bifurcation of the common iliac artery in mice were collected for gross Oil Red O staining. The entire aorta was removed from 4% paraformaldehyde and rinsed in 0.9% normal saline, and the excess fat was completely removed under an anatomical microscope. The mother liquor was prepared as a 1% Oil Red O staining solution. A total of 0.2 g of Oil Red powder (A600395-0050; Sangon Biotech Shanghai Co., Ltd., China) was dissolved in 20 mL of isopropyl alcohol and filtered through a filter paper. The 1% Oil Red O mother liquor was diluted with ultrapure water at a ratio of 3:2 to obtain a 0.6% Oil Red O working solution. The stripped aorta was immersed in 0.6% Oil Red O working solution and incubated at 37 °C for 10 min. The incubated aortas were then rinsed with 75% ethanol (20241014; Sinopharm Chemical Reagent Co., Ltd., China). Once the aortic wall returned to its normal milky white color, it was immediately rinsed with normal saline and then spread out for imaging under a stereoscopic fluorescence microscope (M205FA; Leica, Germany).

### Hematoxylin and eosin, Masson, and Sirius red staining

The roots of the mouse aortas were embedded in paraffin, dehydrated, and sectioned. A hematoxylin and eosin (H&E) staining kit (AH0001; Wuhan Aoxing Biotechnology Co. Ltd., Wuhan, China), Masson staining kit (AH0002; Wuhan Aoxing Biotechnology Co., Ltd., China), and Sirius Red staining kit were used for the staining examinations. Staining was performed according to the manufacturer’s instructions to observe the plaque condition at the aortic root.

### Network pharmacological analysis

Data for *Panax ginseng* C.A. Mey and *Salvia miltiorrhiza* Bge were retrieved through the Traditional Chinese Medicine Systems Pharmacology Database and Analysis Platform (TCMSP), and two oral parameters, bioavailability (OB) and drug likeness (DL), were used to preliminarily screen out activity components and albumen targets with OB ≥ 30% and DL ≥ 0.18. The relevant active ingredients screened from TCMSP and the active ingredients for which no relevant protein targets meeting the requirements were retrieved from this database were first included in the PubChem database to obtain the relevant Simplified Molecular Input Line Entry System (SMILE) nodes. The SwissTarget database was then used to retrieve the action targets of the relevant active ingredients. For the active components of the relevant protein targets that could not be obtained from TCMSP, PubChem was used to obtain the relevant active component action targets, and the active components of the relevant targets that could not be collected were deleted, integrated, and deduplicated. Standard gene names were obtained from the UniProt database. According to the Chinese Pharmacopoeia Commission, the main components of *Panax ginseng* C.A. Mey include ginsenoside Rg_1_, ginsenoside Rg_3_, ginsenoside Re, ginsenoside Rb_1_, and the main component of *Salvia miltiorrhiza* Bge is tanshinone I [12].

With “atherosclerosis” as the key word, atherosclerosis-related targets were obtained from the GeneCards, TTD, and OMIM databases. For the GeneCards database, targets with degree of correlation greater than 1 were selected. The target library for the disease was established by merging the three database targets and removing duplicates. Venn diagrams of the targets of *Panax ginseng* C.A. Mey, *Salvia miltiorrhiza* Bge, and atherosclerosis were drawn using a microbioinformatics platform to identify the common targets of drugs and diseases. Cytoscape software was used to construct a “disease—pathway—drug—active ingredient—target” network diagram, and the CytoNCA plugin was used to calculate the degree values of the nodes. Using the degree value as the screening criterion, the key active components of *Panax ginseng* C.A. Mey and *Salvia miltiorrhiza* Bge that act on atherosclerosis were screened. Common targets were submitted to the STRING database to construct a protein–protein interaction (PPI) network. The minimum interaction threshold was set as “medium confidence (0.400).” The.tsv files were loaded and imported into Cytoscape 3.9.1 for visual analysis. The median of these parameters was used as the screening criterion to identify the key targets of atherosclerosis that *Panax ginseng* C.A. Mey and *Salvia miltiorrhiza* Bge act on. Common targets were subjected to gene ontology (GO) and Kyoto Encyclopedia of Genes and Genomes (KEGG) enrichment analyses using the DAVID platform. The first 20 KEGG signaling pathways were visualized and analyzed using the microbioinformatics platform and SangerBox.

### Molecular docking analysis

Key bioactive components were identified and retrieved in SDF format from the PubChem bioactivity database (https://pubchem.ncbi.nlm.nih.gov/). The SDF files were processed and converted into Protein Data Bank (PDB) format using Open Babel software. Concurrently, the three-dimensional (3D) structures of the critical target proteins were obtained from PDB (http://www.rcsb.org). The receptor and ligand structures were prepared and optimized using PyMOL and AutoDock Tools 1.5.7 software, generating PDBQT files for docking. Molecular docking was performed between the receptors and ligands using AutoDock Vina software. The resulting docking data were imported into PyMOL for visualization and analysis.

### Molecular dynamics simulations

Molecular dynamics simulations were performed using Gromacs 2022. The system was constructed as follows: the Assisted Model Building with Energy Refinement (AMBER) 14SB force field was applied for proteins; the general AMBER force field (GAFF) was applied for small-molecule ligands; and the transferable intermolecular potential with 3 points (TIP3P) model was used for water molecules. The protein and small-molecule ligand files were merged to build a complex simulation system run under periodic boundary conditions. Simulation parameters were set as follows: an integration time step of 2 fs was used; all bonds involving hydrogen atoms were constrained using the linear constraint solver (LINCS) algorithm; electrostatic interactions were calculated using the Particle-Mesh Ewald (PME) method with a cutoff radius of 1.2 nm; and the cutoff value for van der Waals and other non-bonded interactions was set to 10 Å. Temperature and pressure were maintained using the V-rescale thermostat and Berendsen barostat methods, respectively, with target values of 298 K and 1 bar. The system first underwent NVT equilibration for 100 ps at 298 K, followed by 100 ps of NPT equilibration. After equilibration, a 100-ns production simulation was conducted at the same temperature and pressure, and trajectory snapshots were saved every 10 ps. After completion of the simulation, trajectory visualization and analysis were performed using VMD and PyMOL, and the binding free energy between the protein and the small-molecule ligand was calculated using the g_mmpbsa program using the MMPBSA method.

### Western blotting

Radioimmunoprecipitation assay (RIPA) lysis buffer containing a phosphorylase inhibitor (P0013B; Beyotime, China) was added to mouse aortic tissue of a specific weight, and the samples were lysed on ice for 40 min. Next, the proteins were thoroughly homogenized using a tissue grinder, and the supernatant was centrifuged. For cell proteins, the culture medium was removed, and lysis buffer was added at a ratio of 150 μL per well in a 6-well plate. The samples were lysed on ice for 30 min, and the supernatant was centrifuged. The protein concentration in each sample was detected (P0010S; Beyotime, China); protein loading buffer (LT101L; Epizyme Biomedical Technology, China) was added; and the sample was denatured at 95 °C for 5 min.

The total protein sample was used to prepare the gel using the Omni-Easy™ one-step polyacrylamide gel electrophoresis (PAGE) gel rapid preparation kit according to the manufacturer’s instructions (PG212; Epizyme Biomedical Technology, China). The proteins were subsequently transferred onto a polyvinylidene difluoride (PVDF) membrane (IPVH00010; Millipore, USA) and blocked for 10 min with a protein-free rapid blocking solution (PS108P; Epizyme Biomedical Technology, China). Neurogenic locus notch homolog protein 1 (NOTCH1; 10062-2-AP; Proteintech, China), hairy and enhancer of split-1 (HES1; A0925; ABclonal, China), NOD-, LRR- and pyrin domain-containing protein 3 (NLRP3; 30109-1-AP; Proteintech, China), nuclear factor (NF)-κB p65 (10745-1-AP; Proteintech, China), phospho-NF-κB p65 (82335-1-RR; Proteintech, China), and NADPH oxidase 2 (NOX2; 19013-AP; Proteintech, China) were used. The secondary antibodies were a combination of goat anti-rabbit IgG (AS061; ABclonal, China) and goat anti-mouse IgG (SA00001-1; Proteintech, China) linked to the corresponding horseradish peroxidase (HRP) at room temperature. The PVDF membrane was overlaid with Sparkjade ECL super (ED0015-B, SparkJade, China), and the image was presented using a Tanon-5200 system (Shanghai, China). GAPDH (10494-1-AP, Proteintech, China) and β-actin (66009-1 g, Proteintech, China) were used as internal references, and gray scale values were calculated by ImageJ software.

### Immunohistochemistry analysis

The roots of the mouse aorta were paraffin-embedded, dehydrated, dewaxed, rehydrated, and subjected to antigen repair. Antigen repair was performed using 5% bovine serum albumin (BSA), and CD86 (13395-1-AP; Proteintech, China) was diluted at a ratio of 1:800. The secondary antibody was labeled with HRP/AP and incubated at room temperature for 40 min. 3,3′-Diaminobenzidine (DAB) color development was performed under a microscope; hematoxylin counterstaining was performed; and finally, dehydration, transparency, and sealing treatments were performed.

### Cell culture

RAW264.7 cells (CL-0190; Procell, China) were cultured in Dulbecco’s modified Eagle medium (DMEM) supplemented with 10% fetal bovine serum (BL205A; Biosharp, China) and 100 μL/mL penicillin/streptomycin (Beyotime Biotechnology, China) (C11995500BT, GIBCO, China) at 37 °C. Cells were cultured in an incubator with 5% carbon dioxide (Thermo Fisher Scientific).

### Establishment of the cell model

RAW264.7 cells in the logarithmic growth phase were inoculated into 6-well plates (5 × 10^5^ cells/mL). After the cells adhered to the wall, 250 ng/mL lipopolysaccharide (LPS; L861706-5 mg; Macklin, China) and interferon-gamma (IFN-γ; HY-P7071; MCE) were added. After stimulation with 100 ng/mL for 24 h, the expression of the M1-type macrophage markers cyclooxygenase-2 (COX-2; AB188183; Abcam, UK) and inducible nitric oxide synthase (iNOS; AB178945; Abcam) was detected by western blot analysis [14].

### Cell viability assay

Cell viability was determined using a Cell Counting Kit-8 (CCK-8) assay (CT0001-D; Spark Jade, China). The cells in the logarithmic growth phase were inoculated into 96-well plates, with 100 μL of a 1 × 10^4^ cell suspension in each well. Wells with medium containing dimethyl sulfoxide (DMSO; D8371; Solarbio, China) and wells containing only the culture medium without cells were used as controls. After the cells adhered to the wall, different concentrations of ginsenoside Rg_1_ (S33043-1 g; Shanghai Yuanye Bio-Technology Co., Ltd., China) and tanshinone II_A_ (S31459-1 g) were added to the wells. An M1-type macrophage model was established. After 24 h, the medium was discarded. The drug concentrations of ginsenoside Rg_1_ and tanshinone II_A_ were 0, 10, 20, and 30 μg/mL, the optimal dose was selected. After the drug treatment ended, 10 μL of CCK-8 solution was added to each well. The entire procedure was performed in the dark. After 2 h of incubation, the optical density (OD) value of each well was measured at 490 nm. The experiment was repeated three times. The final concentration of ginsenoside Rg_1_ was 20 μg/mL, and that of tanshinone II_A_ was 10 μg/mL.

### Cell grouping

The cells were divided into the control group, model group, GSK2795039 inhibitor group (GSK group) (G882609-1 mg; Macklin, China), and ginsenoside Rg_1_ group and tanshinone II_A_ (RA) group. Cells in the control group did not receive any treatment. The cells in the model group were jointly stimulated with LPS at 250 ng/mL and IFN-γ at 100 ng/mL for 24 h. In the GSK group, after pretreatment with 6 μmol/L for 2 h, 250 ng/mL LPS and 100 ng/mL IFN-γ were added to establish the model. The cells in the RA group were pretreated with ginsenoside Rg_1_ at 20 μg/mL and tanshinone II_A_ at 10 μg/mL for 2 h, after which LPS at 250 ng/mL and IFN-γ at 100 ng/mL were added to establish the model.

### Immunofluorescence assay

RAW264.7 cells (1 × 10^4^ cells/well) were seeded in 24-well plates. After group-specific treatments, the cells were washed twice with PBS and fixed with 4% paraformaldehyde (P1110; Solarbio, China) for 20 min. Permeabilization was performed using 0.1% Triton X-100 (1:1000 dilution in PBS; P1080; Solarbio, China) at room temperature for 30 min. The cells were blocked with donkey serum (EE0009; Spark Jade, China) at 37 °C for 30 min. After serum removal, primary antibodies against COX-2 (66351-1-Ig; Proteintech, China) and iNOS (80517-1-RR; Proteintech, China) diluted 1:800 in PBS were applied, and the cells were incubated overnight at 4 °C. After three PBS washes, the secondary antibodies, Goat Anti-Mouse IgG (H + L) Alexa Fluor 594 (EF0010; SparkJade, China) and Cy3-conjugated Goat Anti-Rabbit IgG (H + L) (AS007; ABclonal, China) were added, and the cells were incubated at room temperature for 30 min. After three additional PBS washes, the samples were mounted with an anti-fade mounting medium containing 4′,6-diamidino-2-phenylindole (DAPI; EE0015; SparkJade, China) for 20 min at room temperature prior to imaging.

### Detection of reactive oxygen species

The reactions were performed in accordance with the reactive oxygen species (ROS) kit instructions (S0033S; Beyotime, China). Cells were inoculated into 24-well plates. After the cells adhered to the surface, the drug was administered and modeling was performed. After 24 h, the cells were washed twice for 30 s each. The pre-configured fluorescent probe was added to a 24-well plate at a volume of 1 mL per well, and the mixture was incubated in the dark. The six-well plate was incubated at 37 °C in the dark for 30 min. After incubation, the plate was washed twice with PBS, and a photograph was obtained.

### Quantitative real-time polymerase chain reaction

Total RNA was extracted from RAW264.7 cells using the SPARKeasy Tissue/Cell RNA Rapid Extraction Kit (with a genomic DNA clearance column) (AC0202-B, SparkJade, China) according to the manufacturer's instructions. The primer sequences were synthesized by Sangon Biotech (Shanghai, China) and are listed in Table 2. Complementary DNA (cDNA) was subsequently synthesized by reverse transcription using 2 × SYBR Green qPCR Mix (with ROX) (AH0104-C, Sparkjade, China). The amount of RNA in the cells was determined using a SPARKscript II RT Plus Kit (with gDNA Eraser) (AG0304-C, Sparkjade, China). The experimental process was as follows: denaturation at 94 °C for 3 min, amplification at 95 °C for 10 s, amplification at 60 °C for 30 s, and return to step 2. This cycle was repeated three times. The relative expression level of the target gene was calculated using the 2^–ΔΔCt^ method (ΔCt = target gene—β-actin, ΔΔ = ΔCTexperiment—ΔCT Control).

**Table 2:** Primer sequence of qRT-PCR assay

Primer name	Forward primer	Reverse primer
notch 1	GCCAGCAAGAAGAAGCGGAGAG	ATTGTCGTCCATCAGAGCACCATC
hes 1	TCACAGCGGCCCGGTCATC	TGAGCGAGGAGCCACTGGAAG
nf-κb	GATGGGACTACACCTCTGCATAT	AGGCTCATACGGTTTCCCATTTA
nlrp3	TATCTCTCCCGCATCTCCATTTG	GCGTTCCTGTCCTTGATAGAGTA
nox2	CTGCCTCCACACCTCCTCTTCC	GGGTTGCCTCATCCAGCCTTG
β-actin	CTCACCATGGATGATGATATCGC	AGGAATCCTTCTGACCCATGC

### Statistical analysis

All statistical analyses were performed using GraphPad Prism 8.0 software. One-way analysis of variance (ANOVA) was used to compare the measurement data that showed a normal distribution, whereas nonparametric tests were used for data that did not conform to a normal distribution. *P* < 0.05 indicated that the difference was statistically significant.

## Results

### Analysis of the active components of ESD

**Fig. 1:**
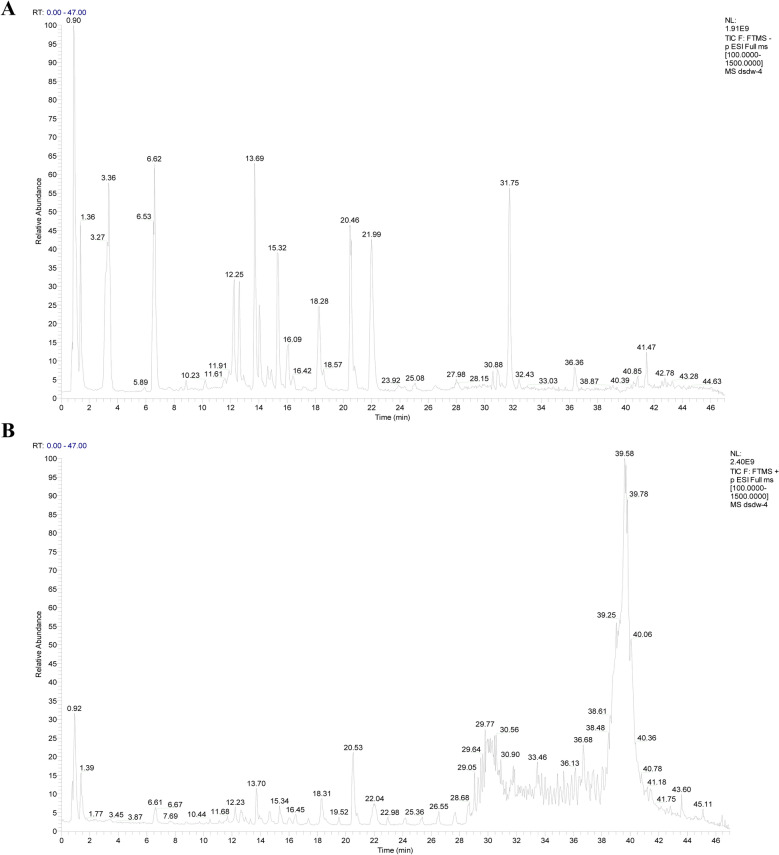
Identification of the chemical constituents of ESD. ESD samples were detected by UPLC− Q-Exactive-MS/MS. ESD samples are shown by total ion chromatography in negative (**A**) and positive (**B**) ion modes

Analysis of active components was performed using a 100 mg/mL ESD solution. Using databases such as TCMSP and PubChem and the related literature for comparison, 20 effective chemical components (Table 3, Figs. 1A, B) that have been found to delay the development of atherosclerosis were identified [15–17].

**Table 3:** 20 chemical constituents of ESD were identified by UPLC-Q-Exactive-MS/MS

NO	Name	RT (min)	Formula	Measured MS (Positive)	Measured MS (Negative)	PPM	MS^2^	Ion mode	PubChem CID
1	(20R)-Ginsenoside Rh1	15.96	C_36_H_62_O_9_	639.44666		− 0.19829	123.11695, 139.11200	M + H	12855920
2	20(S)-Ginsenoside Ck	24.5	C_36_H_62_O_8_	645.43369		3.25520	81.07062, 109.10168	M + Na	9852086
3	Artemisinin	19.7	C_15_H_22_O_5_	305.13446		− 4.84883	165.09108, 83.04988	M + Na	68827
4	Atractylenolide II	22.22	C_15_H_20_O_2_	233.15347		− 0.58802	233.1538, 215.14328	M + H	14448070
5	Calycosin	18.71	C_16_H_12_O_5_	285.07581		0.19526	85.02923, 121.02856	M + H	5280448
6	Formononetin	16.29	C_16_H_12_O_4_	269.08005		− 2.94481	85.02921, 109.02886	M + H	5280378
7	Forsythin	12.38	C_27_H_34_O_11_	535.21735		− 0.08185	136.04762, 137.05954	M + H	24721571
8	Ginsenoside Rg3	21.99	C_42_H_72_O_13_	785.50433		− 0.30109	85.02911, 97.02895	M + H	9918693
9	Glabridin	22.09	C_20_H_20_O_4_	325.14182		− 4.99722	267.10233, 223.07576	M + H	124052
10	Tanshinone II_A_	27.38	C_19_H_18_O_3_	295.13293		0.21878	261.09125, 261.09125	M + H	164676
11	Magnolol	23.73	C_18_H_18_O_2_	267.13821		0.95124	169.06488, 209.09550	M + H	72300
12	Ginsenoside Rb_3_	17.32	C_53_H_90_O_22_		1078.59237	2.51014	313.07236	M + Cl−	12912363
13	Gardenin B	22.32	C_19_H_18_O_7_		357.09842	− 0.11289	995.04863, 77.03792	M−H	96539
14	Ginsenoside Rg_1_	17.24	C_42_H_72_O_14_		799.48492	− 3.16747	475.37952, 179.05556	M + COOH	441923
15	Harpagide	1.65	C_15_H_24_O_10_		363.12967	2.93918	89.02274, 187.11244	M−H	93045
16	Bayogenin	22.56	C_30_H_48_O_5_		487.34369	1.61891	125.09570	M−H	119034
17	Polyphyllin I	23.97	C_44_H_70_O_16_		853.4585	− 0.71932	97.02793, 89.02281	M−H	46173859
18	Eupalinolide A	15.34	C_24_H_30_O_9_		461.17984	− 4.05461	57.03294, 461.23657	M−H	131752313
19	Loureirin B	18.88	C_18_H_20_O_5_		315.12378	− 0.0657	57.03300, 119.04867	M−H	5351344
20	Ferulic acid	7.95	C_10_H_10_O_4_		193.04973	− 4.67031	167.03363	M−H	445858

### ESD significantly attenuated the progression of atherosclerosis in ApoE⁻/⁻ mice

**Fig. 2:**
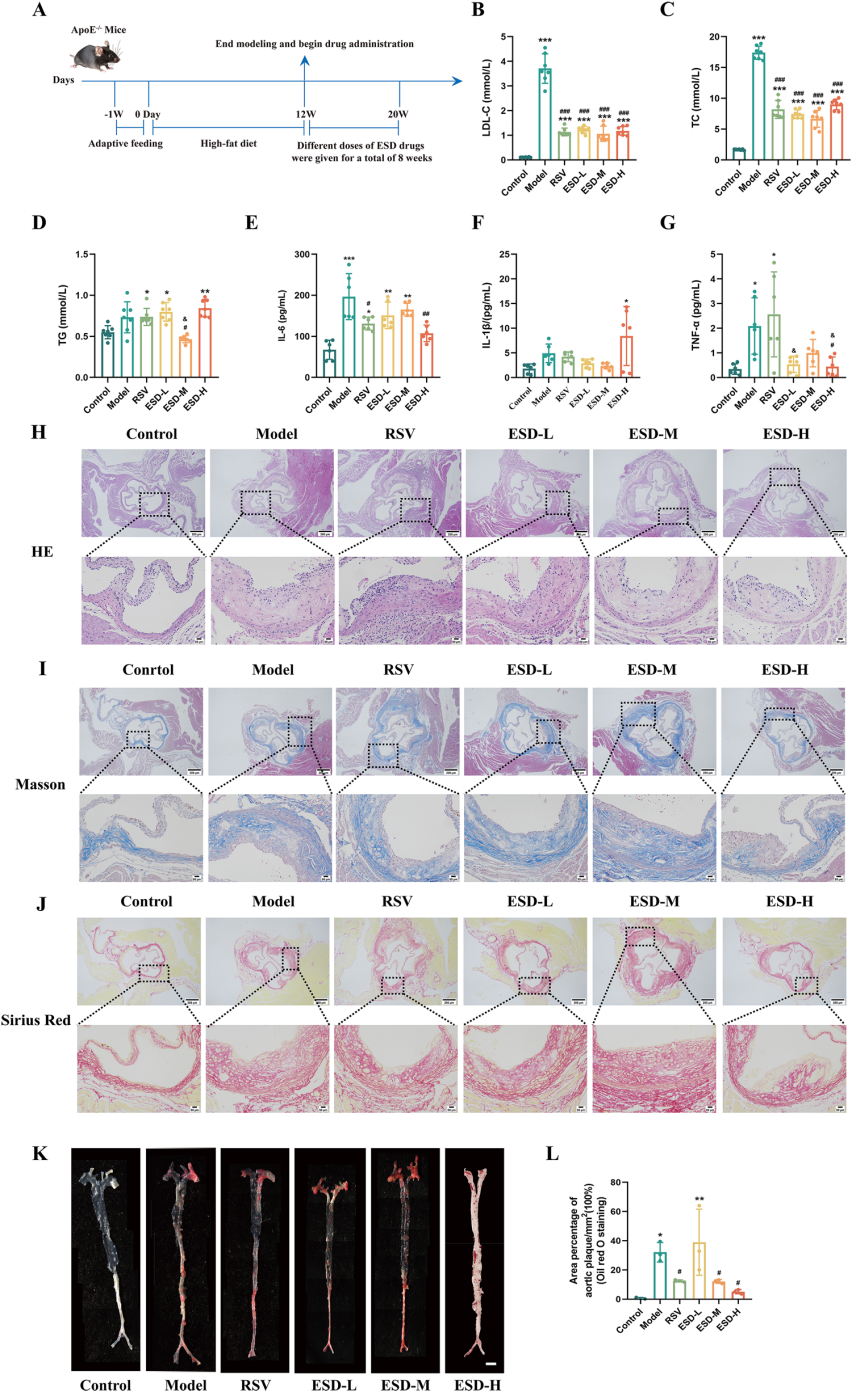
ESD shows therapeutic effects in atherosclerotic mice by inhibiting the secretion of inflammatory factors. (**A**) Schematic diagram of the modeling and handling process of ApoE^−/−^ mice. (**B**–**D**) The LDL-C, TC, and TG levels in mouse serum (n = 7). (**E**–**G**) Expression levels of IL-6, IL-1β, and TNF-α in mouse serum (n = 6). (**H**) HE staining image of the aortic root, scale: 100 μm. **I** Masson staining of aortic plaques, scale: 100 μm. **J** Sirius Red staining image of the aortic root, scale: 100 μm. (**K**) Gross Oil Red O staining of the aorta. scale: 5 mm. (**L**) Analysis of gross Oil Red O staining. Statistical data are expressed as mean ± SD (n = 3). Statistical data are expressed as mean ± SD. In comparison with the control group, ^*^P < 0.05, ^**^P < 0.01, and ^***^P < 0.001. In comparison with the model group, ^#^*P* < 0.05, ^##^*P* < 0.01, and ^###^*P* < 0.001. In comparison with the RSV group, ^&^*P* < 0.05

In this study, ApoE^−/−^ mice were fed atherosclerotic model feed for 12 weeks to establish an atherosclerosis model. Different doses of ESD were administered by continuous gavage for 8 weeks (Fig. 2A). To explore the efficacy of ESD, we measured the serum levels of LDL-C, TG, and TC in the animals. We found that LDL-C levels in all treatment groups were significantly greater than that in the control group (*P* < 0.001) and significantly lower than that in the model group (*P* < 0.001) (Fig. 2B). Similarly, TC levels in all treatment groups were significantly greater than that in the control group (*P* < 0.001) and lower than that in the model group (*P* < 0.001) (Fig. 2C). Furthermore, TG levels in the RSV, ESD-L, and ESD-H groups were significantly greater than that in the control group (*P* < 0.05). The TG levels in the ESD-M group were significantly lower than those in the model group (*P* < 0.05) and the RSV group (*P* < 0.05) (Fig. 2D). We next measured the serum levels of IL-6, IL-1β, and TNF-α in the mice. The IL-6 levels in the model, ESD-L, and ESD-M groups were higher than that in the control group (*P* < 0.01, *P* < 0.001). However, the IL-6 levels in the RSV and ESD-H groups were lower than that in the model group (*P* < 0.05, *P* < 0.01) (Fig. 2E). In comparison with the control group, the ESD-H group showed a significantly greater IL-1β level (*P* < 0.05) (Fig. 2F). In comparison with the control group, the model and RSV groups showed higher serum TNF-α levels (*P* < 0.05). In comparison with the model group, the ESD-H group showed significantly lower TNF-α levels (*P* < 0.05). In comparison with the RSV group, the ESD-L and ESD-H groups showed significantly lower TNF-α levels (*P* < 0.05) (Fig. 2G). In conclusion, these results indicate that ESD reduces blood lipid levels in mice and may inhibit the secretion of inflammatory factors by macrophages by reducing the serum IL-6 and TNF-α levels.

To clarify the effects of ESD on ApoE^−/−^ mice, we conducted HE, Masson, and Sirius Red staining of the root of the mouse aorta (Fig. 2H–J). In the control group, the structure of the aortic root was intact; the intima was not thickened; and no atherosclerotic plaque formation was observed. In the model group, the integrity of the aortic root was disrupted and obvious atherosclerotic plaques were observed. We also conducted gross Oil Red O staining to evaluate the area of lipid plaques (Fig. 2K, L). In comparison with the control group, the model group showed significantly more frequent formation of lipid plaques (*P* < 0.05). The improvement in the ESD-L group was poor. Large areas of plaque formation were also observed (*P* < 0.01). In comparison with the model group, the RSV, ESD-M, and ESD-H groups showed significantly smaller plaque areas (*P* < 0.01), indicating that ESD can effectively improve the aortic plaque area in mice and help delay the progression of atherosclerosis.

### Network pharmacological analysis of ESD and its key components

**Fig. 3:**
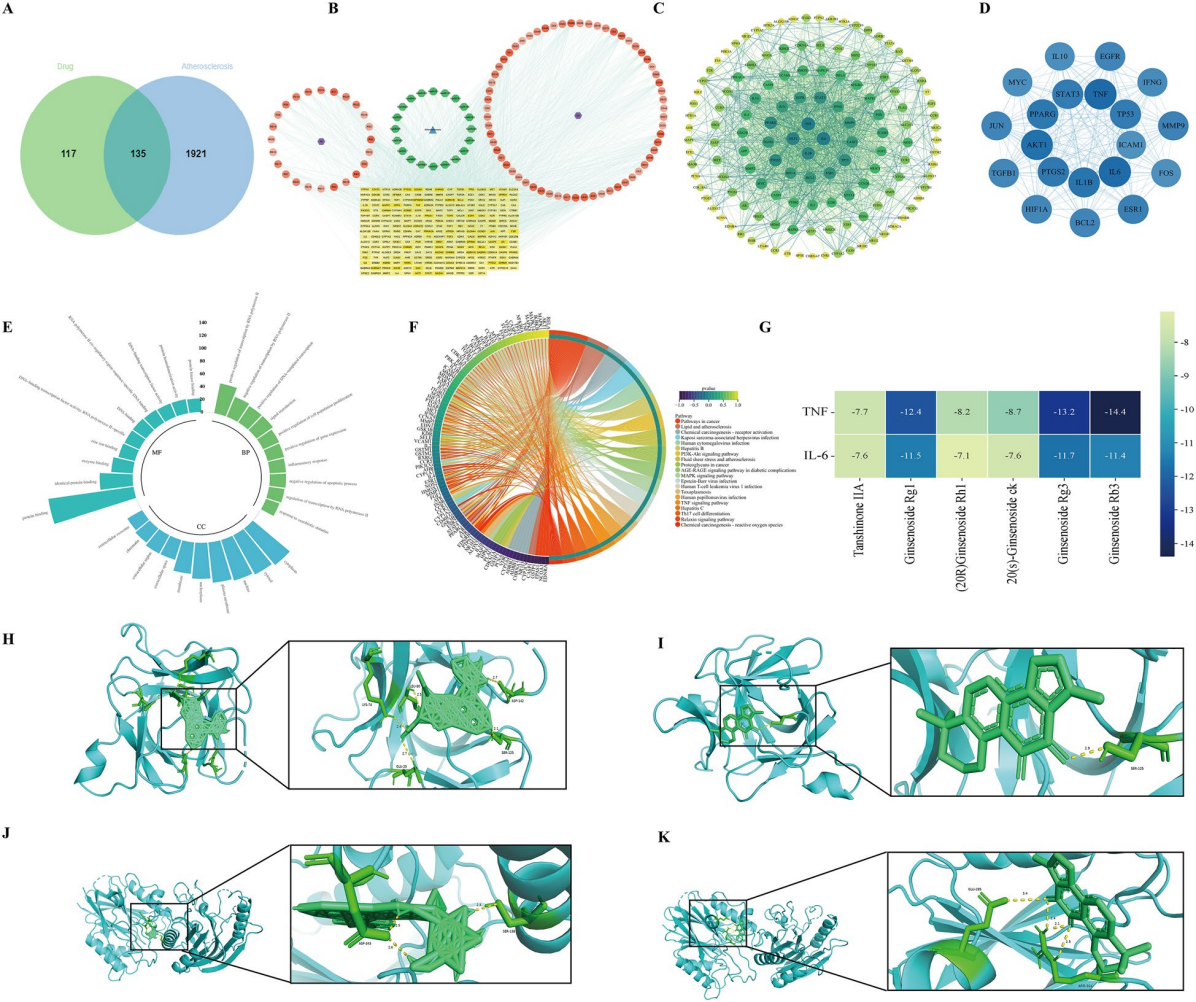
Network pharmacology analysis of key components of ESD. **A** Venny map of potential therapeutic targets for ESD in the treatment of atherosclerotic inflammation. **B** ESD drug-component-target network diagram. Orange represents active ingredients, yellow denotes common targets, green indicates pathways, and blue signifies diseases. **C** PPI network. **D** Bitmaps of the top 20 core targets in the protein–protein interaction network. **E** ESD GO Functional enrichment analysis diagram. **F** Enrichment analysis diagram of the core target KEGG pathway. **G** Visualization analysis. **H** IL-6 and ginsenoside Rg_1_ docking diagram. **I** IL-6 and tanshinone II_A_ docking diagram. **J** TNF-α and ginsenoside Rg_1_ docking diagram. **K** TNF-α and tanshinone II_A_ docking diagram

Screening identified 24 active components from *Panax ginseng* C.A. Mey. and 63 from *Salvia miltiorrhiza* Bge. Using the UniProt database, 252 potential targets of these components were identified. Atherosclerosis-related targets were acquired from the GeneCards (relevance score > 1), TTD, and OMIM databases, yielding 2056, 36, and three targets, respectively. After merging and deduplication, 2056 disease targets remained. Intersecting the drug component targets with disease targets yielded 135 common targets (Fig. 3A). A “Disease-Pathway-Drug-Active Component-Target” network was constructed using Cytoscape, with node color intensity reflecting degree centrality values. Common targets were imported into STRING to build a PPI network (Fig. 3B, C). Using the CytoNCA plugin, six topological parameters, namely, betweenness centrality, closeness centrality, degree centrality, eigenvector centrality, local average connectivity, and network centrality, were calculated for nodes in the PPI network. Targets with all six parameters exceeding their respective median values were selected (49 targets). The parameters were recalculated for these 49 targets, and a second screening that retained targets exceeding all median values identified 20 core targets (Fig. 3D). The results indicated that TNF-α, IL-6, IL-1β, AKT1, among others, were core targets.

Enrichment analysis of 135 common targets was performed using the DAVID database for GO and KEGG pathways (*P* < 0.05). The top 10 significantly enriched Biological Processes, Cellular Components, and Molecular Functions, along with the top 20 enriched KEGG pathways, were visualized (Fig. 3E, F). These pathways, including the TNF, phosphoinositide 3-kinase (PI3K)/protein kinase B (AKT), and mitogen-activated protein kinase (MAPK) signaling pathways, are closely associated with inflammatory responses. Using the UPLC-Q-Exactive-MS/MS results, the major constituents were selected for molecular docking analysis (Fig. 3G). The results indicated that ginsenoside Rg_1_ and tanshinone II_A_ exhibit strong and stable binding activity toward TNF-α and IL-6. Ginsenoside Rg_1_ demonstrated binding affinities of − 7.2 kcal/mol with IL-6 and − 8.1 kcal/mol with TNF-α (Fig. 3H–J), indicating the formation of stable complexes mediated by multiple hydrogen bonds and hydrophobic interactions. Similarly, tanshinone II_A_ showed even stronger binding to these targets, with binding energy values of − 8.6 kcal/mol for IL-6 and − 9.3 kcal/mol for TNF-α (Fig. 3I–K). Thus, ginsenoside Rg_1_ and tanshinone II_A_ may exert their effects primarily through inhibition of TNF-α, given that both active components exhibit significantly stronger binding affinity toward TNF-α.

Our findings confirmed that TNF-α activates NOTCH1 signaling through MAPK, whose downstream effectors HES1/hairy/enhancer-of-split related with YRPW motif protein 1 (HEY1) suppress NLRP3 transcription. IL-1β, released by NLRP3 inflammasome activation, subsequently activates NF-κB and NOTCH1, forming an inflammatory signaling circuit [18–20]. Therefore, to validate the mechanism underlying the therapeutic effects of ESD, this investigation focused on TNF-α to explore the regulatory effect of ESD on the NOTCH1/NF-κB/NLRP3 pathway [21].

### Molecular dynamics simulation analysis of key active constituents in ESD

**Fig. 4:**
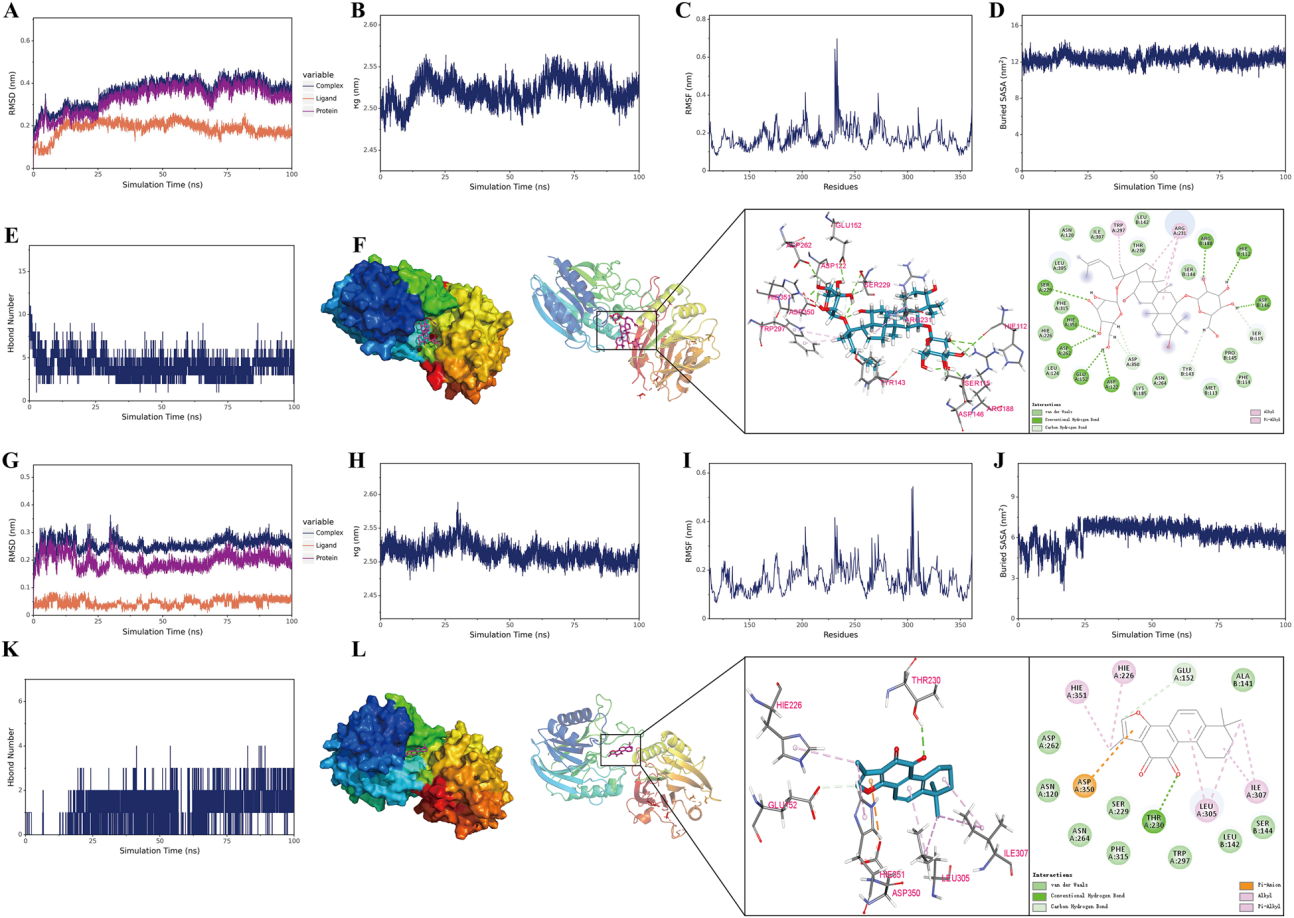
Results of the molecular dynamics simulation. **A** Differences in the RMSD of the plural over time. **B** Rg of ginsenoside Rg_1_ bound to TNF-α. **C** RMSF of TNF-α residues complexed with ginsenoside Rg_1_. **D** Buried SASA at the ginsenoside Rg_1_-TNF-α interface. **E** Hbond number. **F** Interaction energy decomposition for the ginsenoside Rg_1_-TNF-α complex. **G** Differences in the RMSD of the plural over time. **H** Rg of tanshinone II_A_ bound to TNF-α. **I** RMSF of TNF-α residues complexed with tanshinone II_A_. **J** Buried SASA at the tanshinone II_A_ -TNF-α interface. **K** Hbond number. **L** Interaction energy decomposition for the tanshinone II_A-_ TNF-α complex

On the basis of the aforementioned findings, we performed molecular dynamics simulations of ginsenoside Rg_1_ and tanshinone II_A_ complexed with TNF-α. The results demonstrated that both the root mean square deviation (RMSD) and radius of gyration (Rg) of the complexes converged during the simulations (Fig. 4A, B, G, H). Root mean square fluctuation (RMSF) analysis revealed reduced residue flexibility around the ligand-binding site (Fig. 4C and I). A larger buried solvent-accessible surface area (SASA) value indicates stronger intermolecular interactions and a larger contact area. The stabilization of the buried SASA over time suggests that the contact area between the small molecule and the protein became stable (Fig. 4D, J). Collectively, these results suggest that both ginsenoside Rg1 and tanshinone II_A_ can form stable complexes with TNF-α. The number of hydrogen bonds between ginsenoside Rg_1_ and TNF-α remained low, fluctuating between 0 and 1 (Fig. 4E). In contrast, the number of hydrogen bonds between tanshinone II_A_ and TNF-α fluctuated between 0 and 3 (Fig. 4K).

In the analysis of ginsenoside Rg_1_-TNF-α interactions, structural analysis revealed that ginsenoside Rg_1_ formed hydrogen bonds with TNF-α residues SER-229, HIS-351, ASP-262, GLU-152, ASP-122, ASP-146, HIS-112, and ARG-188; showed alkyl and pi-alkyl hydrophobic interactions with ARG-231 and TRP-297; and showed van der Waals interactions with residues such as LEU-142, LEU-124, and PHE-114. Binding energy decomposition demonstrated that van der Waals interactions predominantly contributed to complex stability; electrostatic interactions played a secondary role; and hydrophobic interactions made minor contributions (Fig. 4F). In the analysis of tanshinone II_A_-TNF-α interactions, tanshinone II_A_ formed a hydrogen bond with THR-230 of TNF-α; showed hydrophobic interactions (pi-anion, alkyl, and pi-alkyl) with ASP-350, HIS-351, HIS-226, ILE-307, and LEU-305; and showed van der Waals interactions with ALA-141, ASP-262, and LEU-142 (Fig. 4L).

### ESD inhibits the inflammatory secretion of macrophages through the NOTCH1/NF-κB/NLRP3 signaling pathway and ameliorates atherosclerosis

**Fig. 5:**
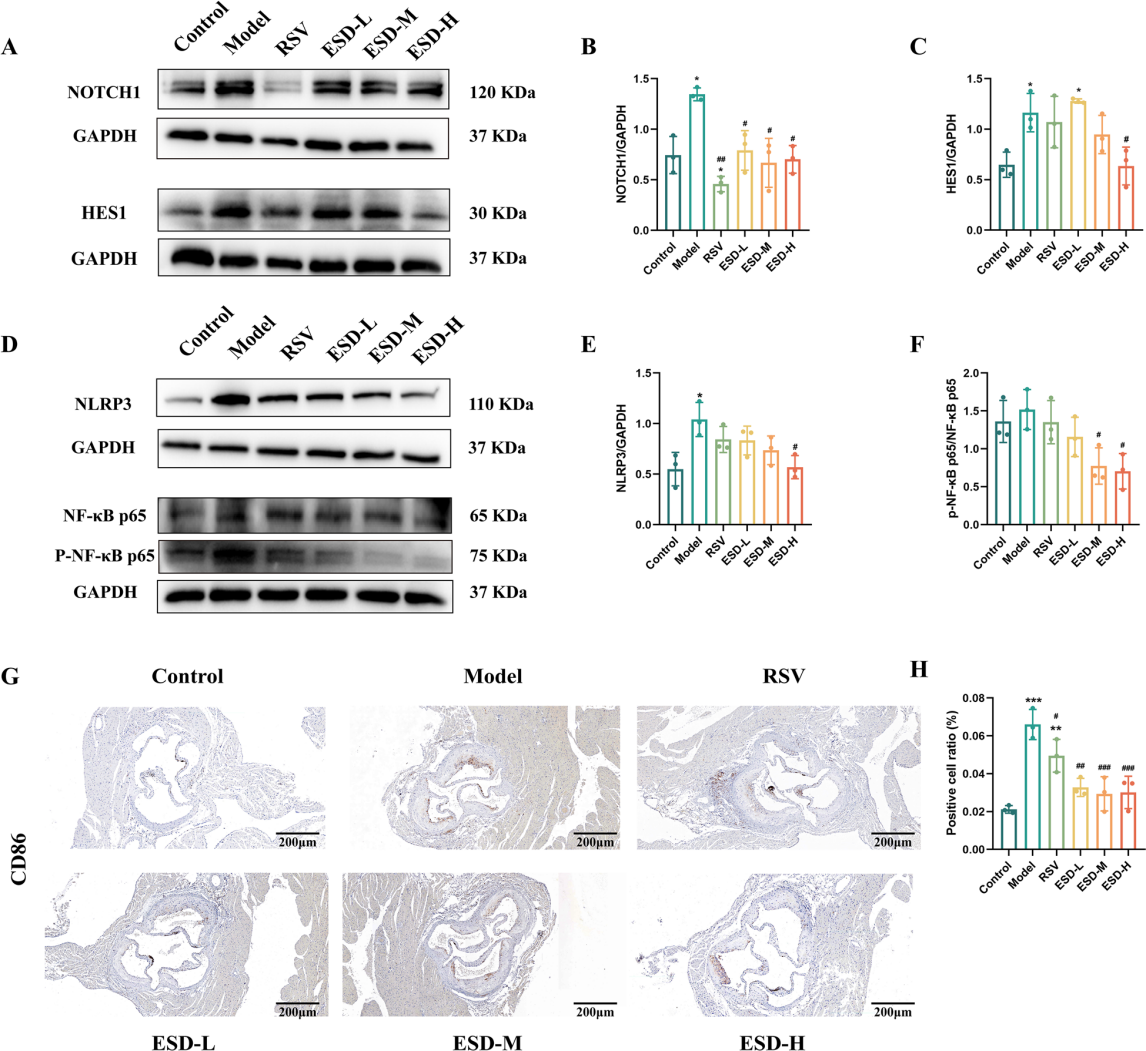
ESD inhibits the NOTCH1/NF-κB/NLRP3 signaling pathway and delays the progression of atherosclerosis. **A** Western blotting analysis was used to detect the protein expression of NOTCH1 and HES1. **B**, **C** Analysis of NOTCH1 and HES1 protein expression. **D** Protein expression of NLRP3, NF-κB p65, and p-NF-κB p65. **E**, **F** Expression analysis of NLRP3, NF-κB p65, and p-NF-κB p65. **G** Immunohistochemical detection of CD86 expression in the aorta of mice, scale: 200 μm. **H** Immunohistochemical statistical analysis (n = 3). Statistical data are expressed as mean ± SD. In comparison with the control group, ^*^P < 0.05, ^***^P < 0.001; in comparison with the model group, ^#^*P* < 0.05, ^##^*P* < 0.01, ^###^*P* < 0.001; in comparison with the RSV group, ^&^*P* < 0.05

On the basis of the results described above, to verify our hypothesis, we first evaluated the protein expression of NOTCH1 and HES1 in the aortas of the mice (Fig. 5A–C). In comparison with the protein expression of NOTCH1 in control group, the expression level in the model group was higher (*P* < 0.05), while that in the RSV group was lower (*P* < 0.05). In comparison with the model group, the RSV, ESD-L, ESD-M, and ESD-H groups showed lower NOTCH1 expression levels (*P* < 0.05, *P* < 0.01, respectively). The protein expression levels of HES1 in the model and ESD-L groups were greater than those in the other groups (*P* < 0.05), while the protein expression level of HES1 in the ESD-H group was significantly lower than that in the model group (*P* < 0.05). Next, we detected the protein expression levels of NLRP3, NF-κB, and p-NF-κB (Fig. 5D–F). In comparison with the control group, the model group showed a higher protein expression level of NLRP3 *(P* < 0.05), whereas in comparison with the model group, the ESD-H group showed a significantly lower protein expression level of NLRP3 (*P* < 0.05). In comparison with the model group, the ESD-M and ESD-H groups showed lower protein expression levels of NF-κB (*P* < 0.05). These results indicate that ESD may delay the progression of atherosclerosis by inhibiting the expression of the NOTCH1/NF-κB/NLRP3 signaling pathway.

Next, we performed immunohistochemical staining of the aortic roots of the mice to evaluate the expression of the M1-type macrophage marker CD86 (Fig. 5G, H). In comparison with the control group, both model and RSV groups showed significant expression of CD86 (*P* < 0.001, *P* < 0.01), indicating that many inflammatory cells accumulated in the aortic roots of atherosclerotic mice, which is consistent with our hypothesis. In comparison with the model group, the ESD-L, ESD-M, and ESD-H groups showed lower expression of CD86 (*P* < 0.01), indicating that ESD inhibited the inflammatory secretion of macrophages.

### The key component of ESD, RA, reduces the expression of ROS in M1-type macrophages through NF-κB and inhibits inflammatory injury

**Fig. 6:**
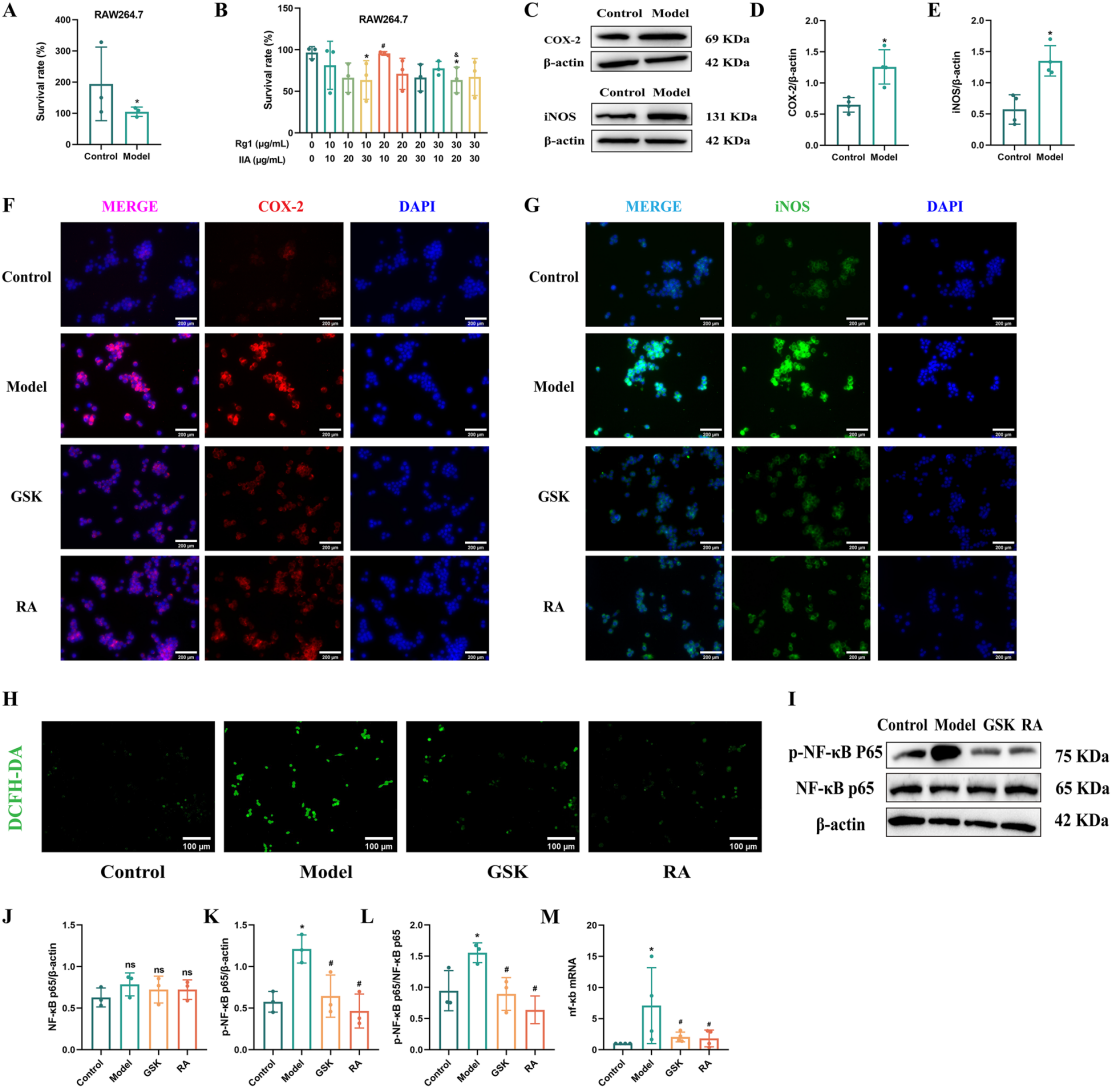
Effects of key components of ESD on M1-type macrophages. **A** The CCK-8 assay was used to detect the cell survival rate after modeling. **B** The CCK-8 assay was also used to detect the cell survival rate of the model after drug pre-protection. **C** Western blotting analysis was used to detect the protein expression levels of COX-2 and iNOS. **D**, **E** Protein expression analysis of COX2 and iNOS (n = 3). **F** COX-2 immunofluorescence staining. Red: COX-2; Blue: DAPI. Scale: 200 μm (n = 3). **G** iNOS immunofluorescence staining. Green: iNOS; Blue: DAPI. scale: 200 μm (n = 3). **H** ROS expression in each group (n = 5), scale: 100 μm. **I** Expression of P65 and p-p65 proteins. **J**–**L** Analysis of P65 and p-p65 proteins (n = 3). **M** Expression of NF-κB mRNA (n = 3). Statistical data are expressed as mean ± SD. In comparison with the control group, ^*^P < 0.05, and in comparison with the model group, ^#^*P* < 0.05

An *in vitro* M1-polarized macrophage inflammation model was established. In combination with data from the Chinese Pharmacopoeia Commission, ginsenoside Rg_1_ and tanshinone II_A_ were identified as the core active components [12]. The CCK-8 assay results demonstrated that cell viability in the ginsenoside Rg_1_ (20 μg/mL) combined with tanshinone II_A_ (10 μg/mL) group (RA group) was significantly higher than that in the other combination groups (*P* < 0.05). Therefore, this combination was selected for subsequent experimental validation (Fig. 6A, B). First, we successfully established an M1-type macrophage model and detected the protein expression of the M1-type macrophage markers COX-2 and iNOS (Fig. 6C). The protein expression levels of COX-2 and iNOS in the model group were significantly higher than those in the control group (*P* < 0.05) (Fig. 6D, E). Additionally, we examined the expression of COX-2 and iNOS using immunofluorescence staining (Fig. 6F, G). The results demonstrated significantly enhanced nuclear translocation in the model group. Both the GSK and RA groups exhibited reduced nuclear fluorescence intensity, indicating that these treatments significantly attenuated the inflammatory response in M1-polarized macrophages. During the inflammatory response, macrophages produce ROS through NADPH oxidase, activating NF-κB to release inflammatory mediators [22, 23]. We speculate that ESD and its active components may cause macrophages to release inflammatory factors by releasing ROS, constantly stimulating intimal damage to the atherosclerotic plaques. Therefore, we selected the NOX2 inhibitor GSK as the positive control and measured the fluorescence intensity of ROS in each group (Fig. 6H). The results revealed that after combined stimulation with IFN-γ and LPS, the ROS level in the model group increased significantly, whereas the ROS levels in both the GSK and RA groups decreased to varying degrees. M1-type macrophages may cause cell loss by generating large amounts of ROS, stimulating the secretion of inflammatory factors, and aggravating atherosclerosis.

To further verify our hypothesis, we first measured the protein expression of NF-κB and p-NF-κB in each group (Fig. 6I–L). In comparison with the control group, the model group showed higher expression levels of both proteins (*P* < 0.05). Moreover, in comparison with the model group, the GSK and RA groups showed lower protein expression levels (*P* < 0.05). We also detected the expression of NF-κB at the genetic level (Fig. 6M). In comparison with the control group, the model group showed significantly greater mRNA levels of NF-κB (*P* < 0.05), and in comparison with the model group, the GSK and RA groups showed significantly mRNA levels of NF-κB (*P* < 0.05).

These results indicate that ESD and its active component, RA, may interfere with the progression of atherosclerosis by reducing the production of ROS, thereby inhibiting the expression of NF-κB and reducing the degree of inflammatory damage to M1-type macrophages.

### The molecular mechanism by which RA, a key component of ESD, regulates M1-type macrophages

**Fig. 7:**
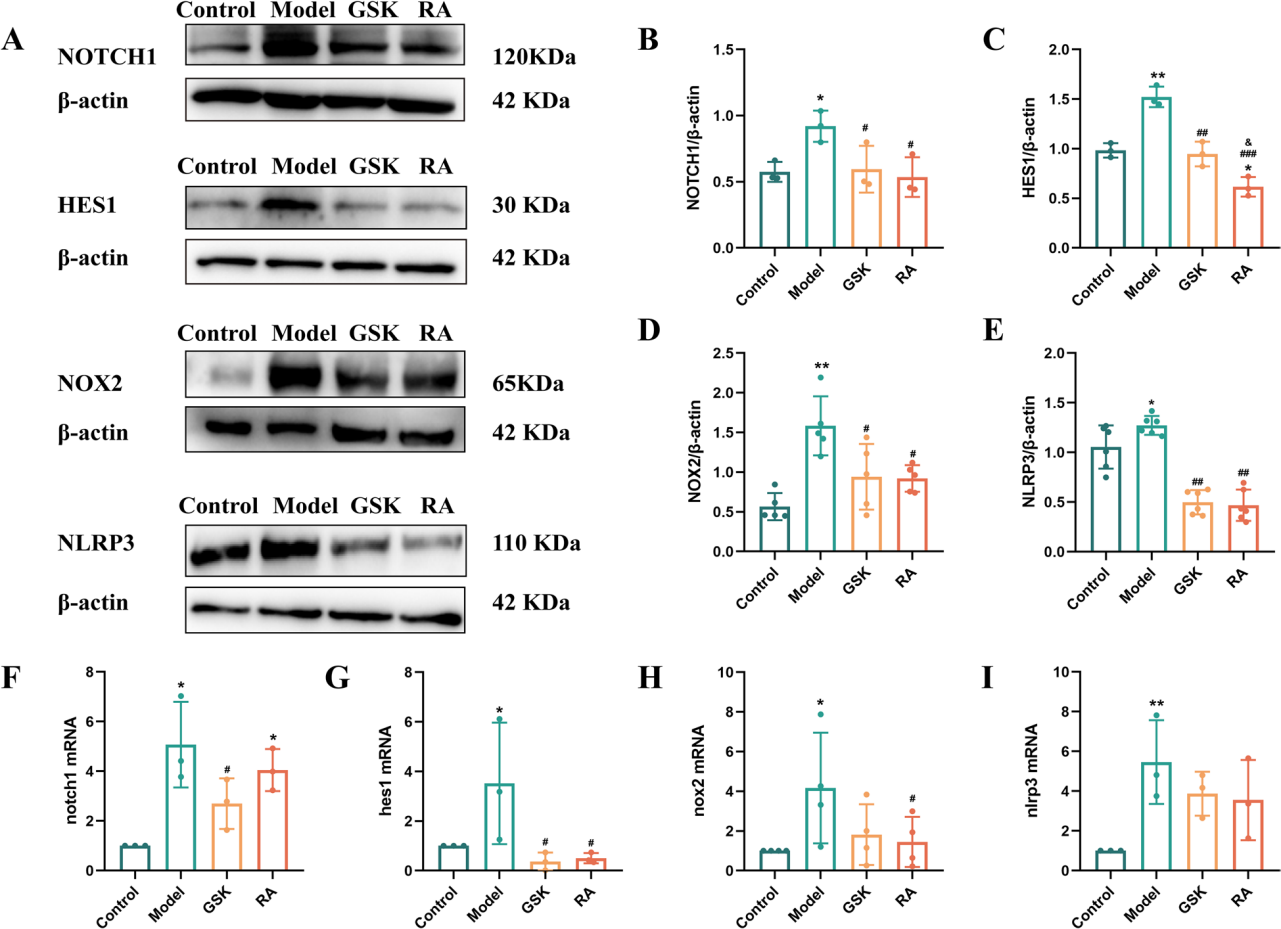
The molecular mechanism by which RA, a key component of ESD, regulates M1-type macrophages. **A** Western blotting analysis was used to detect the protein expression levels of NOTCH1, HES1, NOX2, and NLRP3. **B**–**E** Expression analysis diagrams of NOTCH1, HES1, NOX2, and NLRP3 proteins. **F**–**I** qRT-PCR analysis of the mRNA expression levels of NOTCH1, HES1, NOX2, and NLRP3 in RAW264.7 cells. Statistical data are expressed as mean ± SD (n = 3). In comparison with the control group, ^*^P < 0.05, ^***^P < 0.001; in comparison with the model group, ^#^*P* < 0.05, ^##^*P* < 0.01, ^###^*P* < 0.001; in comparison with the GSK group, ^&^*P* < 0.05

On the basis of the results obtained above, we explored the molecular mechanisms by which RA regulates M1-type macrophages. We measured the protein expression levels of NOTCH1, HES1, NOX2, and NLRP3 (Fig. 7A–E). In comparison with the control group, the model group showed higher protein expression levels of NOTCH1, HES1, NOX2, and NLRP3 (*P* < 0.05, *P* < 0.01). In comparison with the model group, the GSK and RA groups showed significantly lower protein expression levels of NOTCH1, HES1, NOX2, and NLRP3 (*P* < 0.05, *P* < 0.01), and the protein expression level of HES1 in the RS group was lower than that in the GSK group (*P* < 0.05). These findings indicate that RA may exert an inhibitory effect on the secretion of inflammatory factors in macrophages by suppressing the expression of NOTCH, 1HES1, NOX2, and NLRP3 proteins. Next, we measured the expression levels of *notch1*, *hes1*, *nox2*, and *nlrp3* mRNAs Fig. 7F–I. In comparison with the control group, the model group showed higher expression levels of *notch1*, *hes1*, *nox2*, and *nlrp3* mRNAs (*P* < 0.05, *P* < 0.01), while the RA group showed a higher expression level of *notch1* mRNA (*P* < 0.05). In comparison with the model group, the GSK group showed significantly lower expression levels of *notch1* and *hes1* mRNAs (*P* < 0.05), while the RA group showed lower expression levels of *notch1*, *hes1*, and *nox2* mRNAs (*P* < 0.05). These findings indicate that ESD and its main active components inhibit inflammatory secretion of macrophages by suppressing the protein and mRNA expression of NOTCH1, HES1, NOX2, and NLRP3, thereby delaying the progression of atherosclerosis.

## Discussion

Atherosclerosis is a chronic inflammatory disease. Macrophages are pivotal immune cells that are involved in the pathogenesis of this disease. Within susceptible arterial areas, macrophages phagocytose apolipoprotein B-containing lipoproteins (e.g., oxidized LDL), transforming them into lipid-laden foam cells. These foam cells adhere to the vessel wall, recruit inflammatory mediators, and generate ROS, thereby driving plaque formation and progression [8, 24]. M1 macrophages secrete abundant proinflammatory mediators, including cytokines, proteases, and ROS, which sustain vascular wall inflammation. This activity destabilizes vulnerable plaques and shows a negative correlation with plaque stability [25]. Therefore, pharmacological interventions in atherosclerosis may involve shifting macrophage polarization from the proinflammatory M1 phenotype toward the anti-inflammatory M2 phenotype and harnessing their tissue-reparative functions [26]. Experimental studies have confirmed that stimulation with IFN-γ and LPS polarizes resting M0 macrophages into the M1 phenotype, triggering the secretion of proinflammatory factors (e.g., IL-1β, IL-6, TNF-α, and iNOS) that actively drive inflammatory pathogenesis and plaque progression [27]. Notably, this effect was closely related to downregulation of the NOTCH1 signaling pathway. Previous studies have shown that NOTCH1 promotes the differentiation of macrophages into the M1 phenotype by activating downstream target genes such as *Hes1*. Second, excessive activation of NF-κB, the core regulatory factor of the inflammatory response, can induce the assembly of the NLRP3 inflammasome and promote the maturation and release of IL-1β. Thus, the NOTCH1-NF-κB-NLRP3 pathway forms a positive feedback mechanism [28, 29]. Statins and other cholesterol-lowering drugs are commonly used in clinical practice [20]. Moreover, the results of clinical trials have suggested that anti-inflammatory drugs can be used to treat atherosclerosis [30]. Nevertheless, while these drugs can relieve the clinical symptoms of patients, they also have many adverse effects. Therefore, many TCM preparations have been developed to treat this disease.

As a TCM formula, ESD is primarily composed of two herbs, *Panax ginseng* C.A. Mey and *Salvia miltiorrhiza* Bge, and has been proven to possess a variety of biological activities. Studies on the effective components of ESD have revealed that ginsenoside Rb_1_ increases the stability of atherosclerotic plaques by promoting the polarization of anti-inflammatory M2 macrophages, possibly by promoting the expression of IL-4, IL-13, and signal transducer and activator of transcription (STAT) proteins [31]. Ginsenoside Rb_1_ can also increase the stability of atherosclerotic plaques by inducing autophagy in macrophages and reducing lipid accumulation in macrophage foam cells [32]. These findings indicate that the active ingredients of *Panax ginseng* C.A. Mey effectively act on macrophages, exerting an anti-atherosclerotic effect. Several studies have investigated the mechanisms of action of *Salvia miltiorrhiza* Bge. This drug may induce the expression of heme oxygenase (HO)−1 through the PI3K/Akt-MEK1-Nrf2 pathway and reduce the production of intracellular ROS by inducing the expression of HO-1, which has a protective effect on macrophages [33]. Tanshinone II_A_ alleviates atherosclerosis by inhibiting microRNA (miR)−375 to activate KLF4 and enhance autophagy and M2 polarization of macrophages [34]. In conclusion, ESD has shown obvious therapeutic effects in clinical practice. Experimental studies have shown that both *Panax ginseng* C.A. Mey and *Salvia miltiorrhiza* Bge regulate the macrophage phenotype, thereby improving clinical symptoms. However, the mechanism by which ESD and its effective components inhibit M1-type macrophages to exert anti-atherosclerotic effects remains unclear. Therefore, further studies on this topic are warranted.

This study is the first to show that the TCM formula ESD and its active ingredients inhibit the polarization of M1-type macrophages by targeting the NOTCH1/NF-κB/NLRP3 signaling axis, thereby alleviating the inflammatory microenvironment of atherosclerosis. We identified 20 bioactive compounds through UPLC-Q-Exactive-MS/MS analysis of ESD samples. As mentioned above, these compounds afforded protection against vascular endothelial injury, inhibited the polarization of M1-type macrophages, and stabilized plaques. First, we verified the ability of ESD to improve atherosclerosis in ApoE^−/−^ mice. This drug effectively reduced the serum TC, TG, and LDL-C levels as well as the serum IL-6 and TNF-α levels in mice. The ESD-M and ESD-H groups showed the best results. Previous studies have shown that atherosclerosis is an inflammatory disease closely associated with the secretion of inflammatory factors by macrophages. This study explored the mechanism by which ESD exerts its therapeutic effects from the perspective of macrophage inflammation. Therefore, we evaluated the levels of IL-6, IL-1β and TNF-α in the serum of the mice. Macrophages in plaques secrete TNF-α, initiating an early inflammatory response. After the activation of the Toll-like receptor (TLR)/NF-κB pathway, macrophages secrete IL-6, and IL-1β is synthesized by the precursor pro-IL-1β induced by TLR signaling. Caspase-1 is subsequently cleaved into mature IL-1β through the NLRP3 inflammasome [35–37]. In this study, we evaluated the levels of these three inflammatory factors were detected, and our findings revealed that in comparison with the corresponding level in the control group, the IL-6 levels in the model, ESD-L, and ESD-M groups were greater. This may have occurred because establishment of the mouse model was followed by continuous secretion of inflammatory factors by macrophages. Although administration of the model and test drugs decreased the secretion of these factors, the secretion could not be reduced to normal levels. Moreover, in comparison with the control group, the ESD-H group showed significantly higher IL-1β levels, which could be attributed to the tissue damage caused by persistent atherosclerotic plaques and the cellular stress induced by high-dose drug treatment [38].

HE, Masson staining, Sirius scarlet, and gross Oil Red O staining revealed that ESD effectively improved the area of aortic plaques in mice. IL-6 and TNF-α are markers of M1-type macrophages. Therefore, we speculated that ESD may reduce the inflammatory levels of M1-type macrophages. Combining the results of network pharmacology analyses and molecular dynamics simulations, we found that ESD may inhibit inflammatory secretion through the NOTCH1/NF-κB/NLRP3 signaling pathway. Since these key targets and signaling pathways are closely related to inflammation in atherosclerotic diseases, the key factor that induces inflammation is the transformation of macrophages into M1-type macrophages after injury. Increased CD86 content in local proinflammatory plaques is associated with increased plaque vulnerability, indicating that the progression of atherosclerosis is regulated by inflammatory factors [39]. To further verify our hypothesis, we performed immunohistochemical staining of the aortic plaques of mice and evaluated the CD86 content in the different groups. The results revealed that many inflammatory cells accumulated at the root of the aorta in the model group, whereas the expression of CD86 decreased in the ESD-L, ESD-M, and ESD-H groups. These findings indicate that ESD has the potential to reshape macrophages and intervene in atherosclerosis by reducing the number of inflammatory M1-type macrophages in the aortic root.

Progression of atherosclerosis is closely linked to inflammation. The related molecular mechanisms include ROS-dependent signaling pathways, Toll-like receptors, and the NF-κB and Notch pathways. These signaling pathways mostly exert anti-atherosclerotic effects by inhibiting the secretion of inflammatory factors [40, 41]. This finding is consistent with the results mentioned above. TCM regulate macrophage polarization through the synergistic effects of multiple components and targets. On the basis of these previous studies, we investigated the effects of ginsenoside Rg_1_ on atherosclerosis. Using a combination of analyses based on UPLC-Q-Exactive-MS/MS, network pharmacology, molecular dynamics simulations, and the Chinese Pharmacopoeia, we selected the key components of *Salvia miltiorrhiza* Bge and *Panax ginseng* C.A. Mey, tanshinone II_A_ and ginsenoside Rg_1_, as the representative active components of ESD to explore the mechanisms underlying their effects on atherosclerosis [42, 43]. At the cellular level, we further investigated the mechanisms of the validated key active constituents using GSK as the positive control. This is because NOX2 is the primary source of ROS in macrophages, and it also plays a crucial role in the development of atherosclerosis. Studies have shown that the NOX2-specific inhibitor GSK can inhibit ROS production and NADPH consumption, thereby preventing the formation of vulnerable plaques [44]. We established an inflammatory injury model of M1-type macrophages *in vitro* and stimulated RAW264.7 cells with LPS and IFN-γ for 24 h. RA attenuated inflammatory injury in M1 macrophages by suppressing ROS production and downstream NF-κB activation. This reduction consequently inhibited the expression of M1 polarization markers (iNOS and COX-2). Therefore, we examined the effects of RA on the key regulators of macrophage inflammation: protein and mRNA expression of NOTCH1, HES1, NOX2, and NLRP3.

**Fig. 8:**
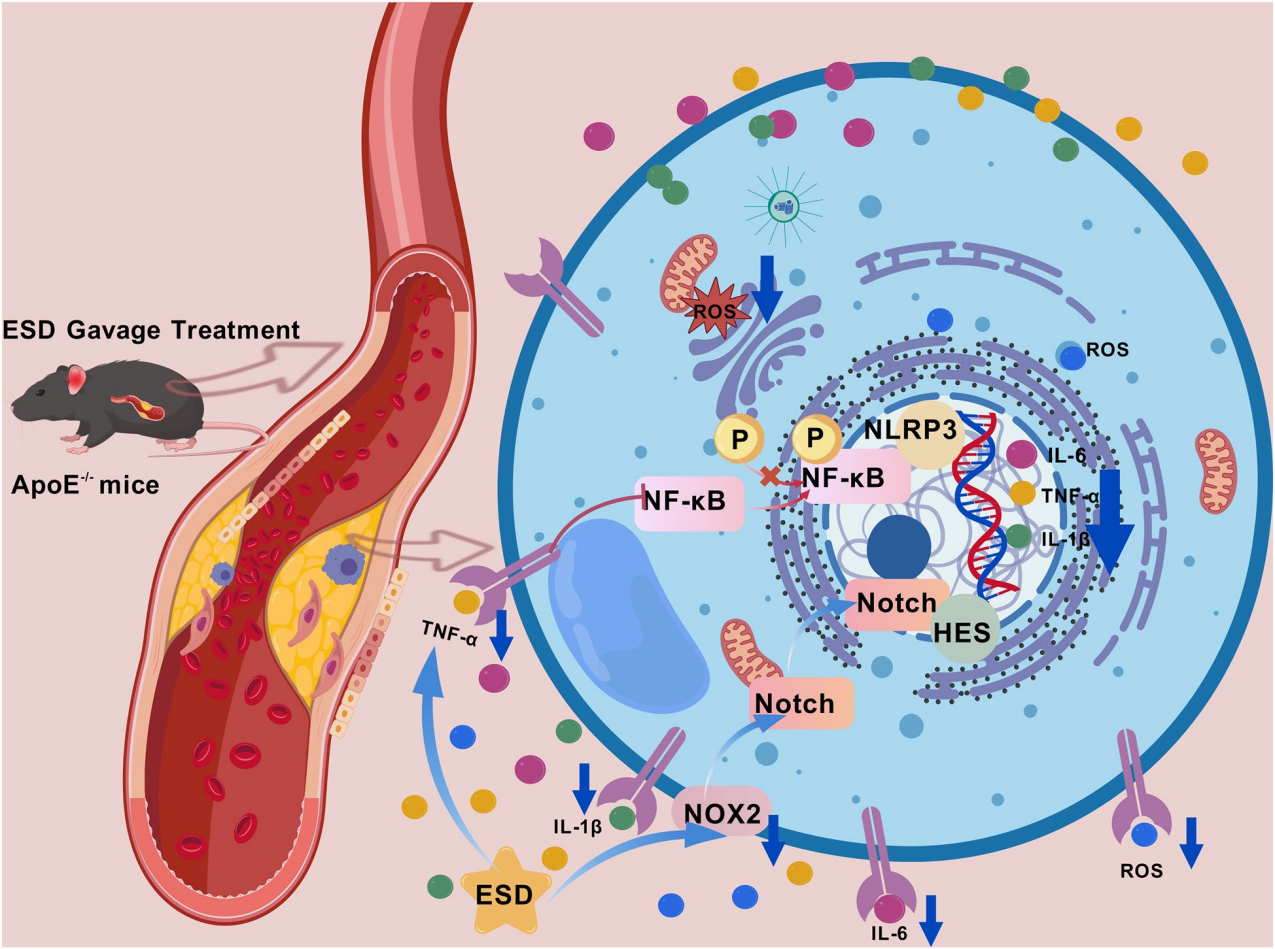
Schematic diagram of ESD intervention in atherosclerosis through the NOTCH1/NF-κB/NLRP3 signaling pathway (Created with BioGDP.com). NOTCH1 promotes macrophage differentiation toward the M1 phenotype by activating downstream target genes such as *Hes1*; NF-κB overactivation induces NLRP3 inflammatory vesicle assembly and promotes IL-1β maturation and release; and NOX2 is a major source of ROS in macrophages. These pathways collectively regulate inflammatory state and ROS levels, and ESD blocked the NOTCH1-NF-κB- NLRP3 signaling pathway circulation

In conclusion, this is the first study to demonstrate that ESD and its active ingredients regulate the NOTCH1/NF-κB/NLRPP3 signaling pathway, reduce the expression of ROS in macrophages, and inhibit the secretion of inflammatory factors, thereby treating atherosclerosis (Fig. 8) [45]. However, this study had several limitations. First, the components of TCM formulations are complex and diverse. This study selected only two key components, and the synergistic effects of the other key components need to be further explored. Second, the mechanisms underlying the therapeutic effects could not be fully elucidated, and the studies of active ingredients are limited to the cellular level. However, the safety and efficacy of these ingredients should be further verified *in vivo*. Finally, relatively few clinical studies related to these two active ingredients exist, and few clinical trials have verified their safety.

## Conclusion

In summary, ESD and its active ingredients can treat atherosclerosis by regulating the NOTCH1/NF-κB/NLRPP3 signaling pathway, reducing the expression of ROS in macrophages and inhibiting the secretion of inflammatory factors. This study provided experimental evidence for the use of ESD in the treatment of atherosclerosis.

## Data Availability

No datasets were generated or analysed during the current study.

## Abbreviations

ESD: Ershen Dan
LDL: Low-density lipoprotein
TG: Triglyceride
TC: Total cholesterol
TCM: Traditional Chinese medicine
UPLC: Ultra performance liquid chromatography
ROS: Reactive oxygen species
MD: Molecular dynamics simulations
RMSD: Root mean square deviation
Rg: Radius of gyration
RMSF: Root mean square fluctuation
Buried SASA: Buried solvent accessible surface area
PME: Particle-Mesh Ewald
WB: Western blot
RT-qPCR: Real-time fuorescence quantitative PCR
CCK-8: Cell counting Kit-8
ELISA: Enzyme-linked immunosorbent assay
TNF-α: Tumor necrosis factor-alpha
IL-6: Interleukin-6
IL-1β: Interleukin-1 beta
H&E: Hematoxylin–Eosin staining
TCMSP: Traditional Chinese medicine systems pharmacology database and analysis platform
OB: Oral bioavailability
DL: Drug likeness
ESD-L: Er Shen Dan Low dose group
ESD-M: Er Shen Dan medium dose group
ESD-H: Er Shen Dan high dose group
RSV: Rosuvastatin
NOTCH1: Neurogenic locus notch homolog protein 1
HES1: Hes family BHLH transcription factor 1
NLRP3: NLR family, pyrin domain containing protein 3‌
NF-κB: Nuclear factor kappa-light-chain-enhancer of activated B cells
COX-2: Cyclooxygenase-2
iNOS: Inducible nitric oxide synthase
IFN-γ: Interferon-gamma
LPS: Lipopolysaccharide
DMSO: Dimethyl sulfoxide
GSK: GSK2795039
RA: Ginsenoside Rg_1_ and tanshinone II_A_
NADPH: Nicotinamide adenine dinucleotide phosphate
